# Identifying Crucial Biomarkers in Osteoporosis and Ulcerative Colitis Through Bioinformatics Analysis of Co-expressed Genes

**DOI:** 10.7759/cureus.45063

**Published:** 2023-09-11

**Authors:** Zhengyan Wang, Xukai Wang, Jing Yan, Ying Wang, Xingxing Yu, Yanpeng Wang

**Affiliations:** 1 Department of Orthopedics, Changchun University of Chinese Medicine, Changchun, CHN; 2 College of Medicine, Changchun University of Chinese Medicine, Changchun, CHN

**Keywords:** ulcerative colitis (uc), gene expression omnibus (geo) database, differentially expressed genes (degs), computational biology, osteoporosis (op)

## Abstract

Osteoporosis (OP) and ulcerative colitis (UC), prevalent immune diseases, exert a substantial socioeconomic impact globally. This study identifies biomarkers for these diseases, paving the way for in-depth research. Initially, the Gene Expression Omnibus (GEO) database was employed to analyze datasets GSE35958 and GSE87466. This analysis aimed to pinpoint co-expression differential genes (DEGs) between OP and UC. Subsequently, the Metascape database facilitated the Gene Ontology (GO) and Kyoto Encyclopedia of Genes and Genomes (KEGG) analysis of these DEGs' co-expression. For network construction and visualization, the STRING11.5 database along with Cytoscape 3.7.2 (Cytoscape Team, USA) were utilized to create a protein-protein interaction (PPI) network. Moreover, Cytoscape’s cytoHubba plugin was instrumental in identifying the central genes, known as hub genes. In the datasets GSE35958 and GSE87466, 156 co-expressed DEGs were discovered. The PPI network, constructed using STRING11.5 and Cytoscape 3.7.2, comprises 96 nodes and 222 connections. Notably, seven hub genes were identified, namely COL6A1, COL6A2, BGN, NID1, PLAU, TGFB1, and PLAUR. These DEGs were predominantly enriched in pathways such as extracellular matrix organization and collagen-containing extracellular matrix, as per GO analysis. For diagnostic model construction and hub gene validation, datasets GSE56815 and GSE107499 from the GEO database were employed. The top five hub genes were validated. In conclusion, the hub genes identified in this study played a significant role in the early diagnosis, prevention, and treatment of OP and UC. Furthermore, they provide fresh insights into the underlying mechanisms of these diseases’ development and progression.

## Introduction

Osteoporosis (OP), the most prevalent metabolic bone disorder, is marked by a continuous loss of bone mass and tissue deterioration [1]. The bone remodeling process is out of balance, resulting in a decrease in bone strength and fractures [2]. OP is common in elderly and postmenopausal women, and the pathogenesis factors and mechanisms are mainly related to the immune system, genetics, estrogen levels in the body, smoking, and other lifestyles [3]. OP is a silent bone disease that is difficult to diagnose and treat in a timely manner because there are often no symptoms until low-energy fractures occur.

Mucous membrane inflammation is the hallmark of ulcerative colitis (UC), a chronic immune-mediated, recurrent inflammatory bowel disease (IBD) [4]. This inflammation begins in the rectum and, in severe cases, extends throughout the colon [5]. The etiology and mechanism of pathogenesis are unknown but mainly involve environmental, immune system, and other factors. Clinical symptoms are mainly abdominal pain, diarrhea, and rectal discharge of pus. In addition, extraintestinal manifestations related to the eyes, liver, and joints may be present [6]. If left untreated, it can lead to persistent bowel damage and increase the risk of surgery [7]. The incidence and prevalence of OP and UC are increasing year on year globally, creating a serious socioeconomic burden and consuming significant medical resources. Therefore, we need to pay attention to it.

OP and UC share commonalities in inflammation, immunity, and other factors, suggesting the possibility of shared or similar core genes, pathways, or biological processes. While previous studies have touched upon related aspects, there remains a gap in understanding the specific correlation between the two diseases. This study aims to bridge this gap by identifying differential genes and potential biomarkers through bioinformatics analysis, which could be instrumental in the prevention and treatment of OP and UC. Four datasets, namely GSE35958 [8], GSE87466 [9], GSE56815 [10], and GSE107499 were downloaded from the Gene Expression Omnibus (GEO) database. The first two datasets, GSE35958 and GSE87466, were employed for identifying co-expressed differential genes, while GSE56815 and GSE107499 were utilized for constructing disease diagnosis models. The analysis began with Gene Set Enrichment Analysis (GSEA) of GSE35958 and GSE87466. Subsequently, the GEO2R tool within the GEO database was used to filter out differentially expressed genes. Separate Gene Ontology (GO) and Kyoto Encyclopedia of Genes and Genomes (KEGG) analyses were conducted. The Venn 2.0 website facilitated the identification of co-expressed differential genes, which were then subjected to enrichment analysis. Utilizing Cytoscape 3.7.2 (Cytoscape Team, USA), the data was visualized, and a protein-protein interaction (PPI) network of co-expressed differential genes was constructed using the STRING (Search Tool for the Retrieval of Interacting Genes/Proteins) database. The cytoHubba plugin within Cytoscape 3.7.2 was employed to pinpoint the Hub genes. Lastly, Statistical Package for the Social Sciences (IBM SPSS Statistics for Windows, IBM Corp., Version 25.0, Armonk, NY) was used to construct a disease diagnostic model, which aided in validating the Hub genes. The identification of Hub genes for OP and UC through this study is expected to offer fresh perspectives for subsequent research and elucidate the biological mechanisms involved.

## Materials and methods

Construction of flowchart and technical route

**Figure 1:**
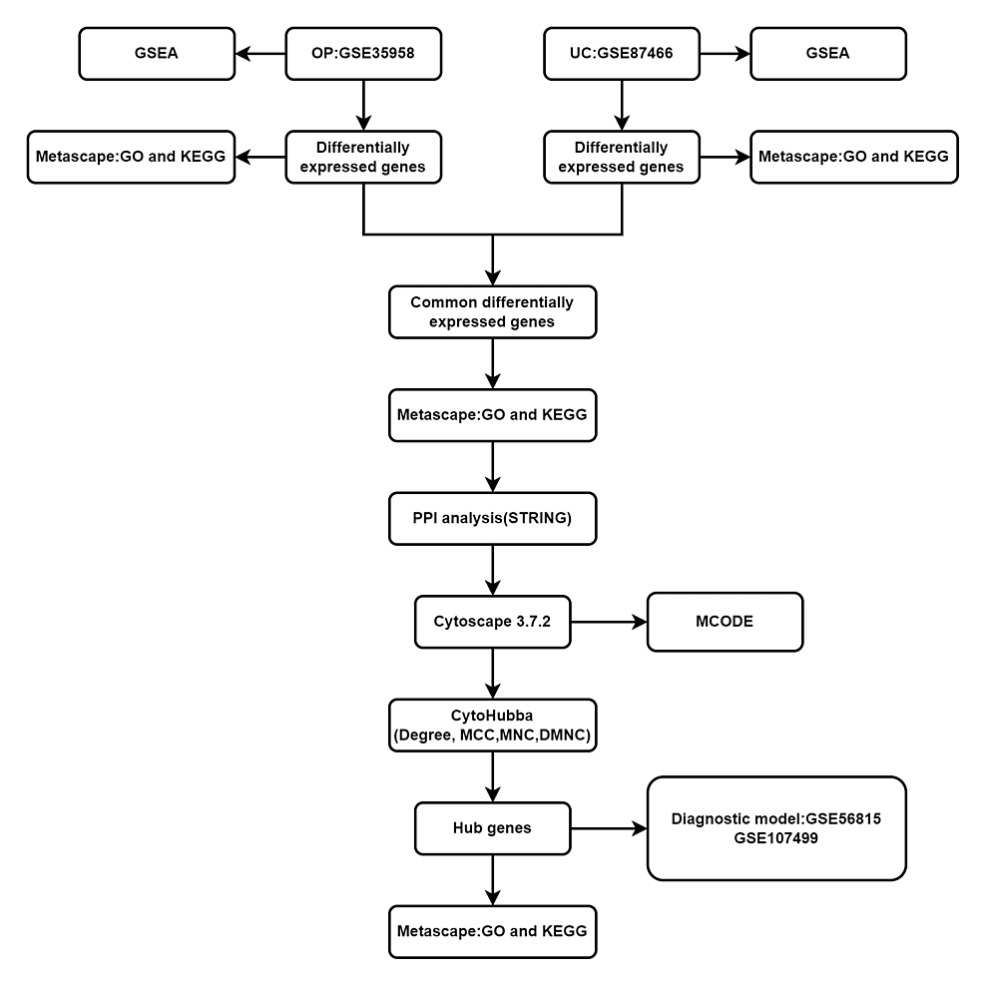
Flow chart GSEA: Gene Set Enrichment Analysis, OP: osteoporosis, UC: ulcerative colitis, KEGG: Kyoto Encyclopedia of Genes and Genomes, GO: Gene Ontology, PPI: protein-protein interaction

We first produced the specific flowchart and technical route of this study shown in Figure 1. We downloaded the GSE35958 (OP) and GSE87466 (UC) datasets from the GEO database and conducted separate GSEA analyses on each of them. Subsequently, we performed differential expression analysis and functional enrichment analysis for the expression profiles of these two datasets. Next, GO and KEGG enrichment analyses were carried out based on the differentially expressed genes shared between the GSE35958 (OP) and GSE87466 (UC) datasets, and a PPI network graph was constructed using these differentially expressed genes. Additionally, we employed the cytoHubba plugin in Cytoscape 3.7.2 to identify hub genes and conducted enrichment analysis on these hub genes. Finally, we validated the hub genes using external independent datasets, GSE56815 (OP) and GSE107499 (UC), to ascertain the accuracy and reliability of our findings.

Data source

The GEO database is utilized to store quality articulation datasets. We downloaded four datasets from GEO, namely GSE35958, GSE87466, GSE56815, and GSE107499 (Table 1). The GPL570 platform ([HG-U133_Plus_2] Affymetrix Human Genome U133 Plus 2.0 Array) serves as the foundation for GSE35958. The GPL13158 platform ([HT_HG-U133_Plus_PM] Affymetrix HT HG-U133+ PM Array Plate) serves as the foundation for GSE87466. The GPL96 platform ([HG-U133A] Affymetrix Human Genome U133A Array) serves as the foundation for GSE56815. The GPL15207 platform, ([PrimeView] Affymetrix Human Gene Expression Array), is the foundation of GSE107499.

**Table 1:** Datasets from the GEO database GEO: Gene Expression Omnibus, OP: osteoporosis, UC: ulcerative colitis, BMD: bone mineral density

Data set	Expression	Platforms	References
GSE35958 [8]		GPL570	PMID:23028809
OP	5		
Normal	4		
GSE87466 [9]		GPL13158	PMID:29401083
UC	28		
Normal	21		
GSE56815 [10]		GPL96	PMID:29330445
Low BMD	21		
High BMD	20		
GSE107499		GPL15207	None
UC	34		
Normal	22		

Enrichment analysis of OP and UC

GSEA is a computational method employed to determine the statistical significance of predefined sets of genes. This approach is particularly useful in identifying subtle changes in gene expression that may be overlooked by other methods. Another essential tool in the analysis is GO, which is widely used to categorize genes based on their biological processes (BP), cellular components (CC), and molecular functions (MF). Additionally, the KEGG is a comprehensive database that contains a wealth of information on genomes, biological pathways, diseases, drugs, and chemical substances. KEGG is particularly renowned for its pathway maps representing molecular interactions and reaction networks. We used the GSEA database and the Metascape database for GSE35958 and GSE87466 for GSEA enrichment analysis and GO enrichment analysis.

Identify co-expressed differential genes

To perform differential genetic analysis and download the data, we make use of the GEO2R tool in the GEO database. With a standard for dataset organization | logFC | ≥ 1.0 and adjusted P Value < 0.05. Co-expressed differential genes were found using the Venn 2.0 database. The Metascape database was used for the subsequent GO and KEGG enrichment analyses, and an adjusted P value < 0.05 was considered statistically significant.

PPI network construction of differentially expressed genes

The revealing of PPI networks can identify core protein genes and reveal both specific and non-specific interactions between proteins. Using the STRING 11.5 database, functional relationships between proteins were predicted, treating PPI networks of differential genes with scores of ≥ 0.4 as functional links, and building visual networks using Cytoscape 3.7.2. Following that, we identified and constructed densely connected modules from the PPI network by utilizing the MCODE plugin in the Cytoscape 3.7.2 software, which has a standard K-core of 2, a degree cutoff of 2, a maximum depth of 100, and a node score cutoff of 0.2. The module's genes were then subjected to enrichment analyses using GO and KEGG.

Selection and analysis of hub genes

In this study, the cytoHubba plugin within the Cytoscape 3.7.2 software was employed to pinpoint hub genes, which are genes that hold central positions within the PPI networks and are often key players in biological functions. Four algorithms, namely Degree, DMNC, MCC, and MNC, were utilized to select the top 20 hub genes. These algorithms are effective in identifying genes with high connectivity and centrality within the network, which are often indicative of their importance in biological processes. Subsequently, Venn 2.0 was used to identify the overlapping genes that were common across all four algorithms. This step is crucial in ensuring that only the most consistently significant genes are considered for further analysis. Finally, GO and KEGG enrichment analyses were conducted on these selected genes. This enrichment analysis helps in understanding the biological processes, cellular components, molecular functions, and pathways in which these hub genes are involved. By employing a combination of network analysis and enrichment studies, this approach provides a comprehensive understanding of the key genes and their roles in the context of OP and UC.

Validation and construction of diagnostic models

In this study, datasets GSE56815 and GSE107499 were employed as validation datasets. The characteristics of the hub genes, particularly those with scores in the top five, were used to generate corresponding expression profiles. This step is crucial for validating the significance and reliability of the identified hub genes. SPSS 25.0, a widely used statistical analysis software, was then utilized to analyze the model's Receiver Operating Characteristic (ROC) and the Area Under the Receiver Operating Characteristic (AUC). The ROC curve is a graphical representation that illustrates the diagnostic ability of a binary classifier system as its discrimination threshold is varied. The AUC represents the degree or measure of separability, indicating how well the model is capable of distinguishing between classes. An AUC value close to 1 indicates that the model has good class separation capabilities, whereas an AUC value close to 0.5 suggests that the model is no better than random guessing. Analyzing the ROC and AUC is essential for evaluating the performance and predictive accuracy of the diagnostic model built on the hub genes.

## Results

Enrichment analysis of OP and UC

**Figure 2:**
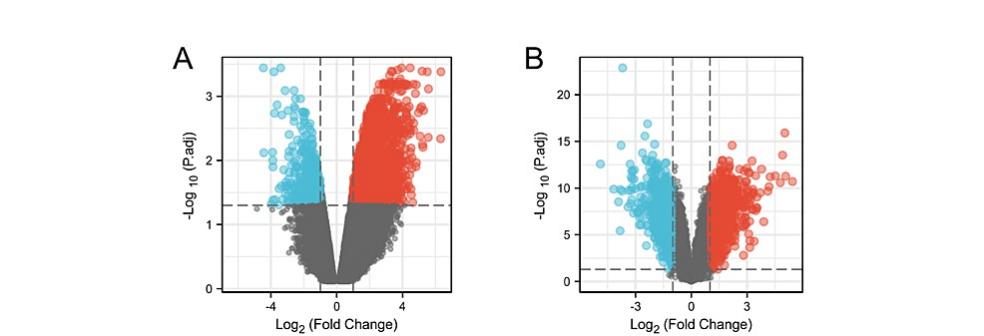
Screening the datasets for differentially expressed genes. (A) A volcanic map of the differential genes identified in the GSE35958 (OP) dataset. (B) A volcanic map of the differential genes identified in the GSE87466 (UC) dataset. OP: osteoporosis, UC: ulcerative colitis

**Figure 3:**
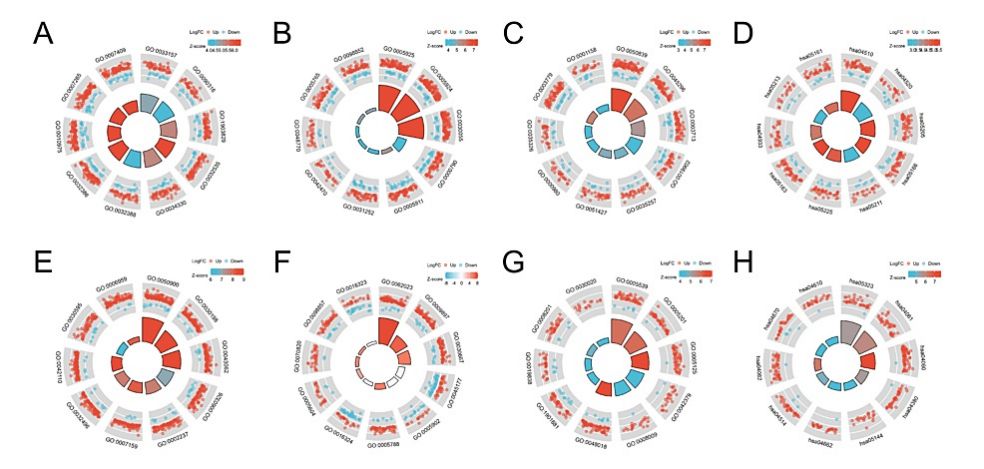
GO and KEGG analysis of the datasets. (A) BP, (B) CC, (C) MF, and (D) KEGG of the GSE35958 (OP) dataset. (E) BP, (F) CC, (G) MF, and (H) (KEGG) of the GSE87466 (UC) dataset. BP: biological processes, CC: cellular components, MF: molecular function, KEGG: Kyoto Encyclopedia of Genes and Genomes, GO: Gene Ontology, OP: osteoporosis, UC: ulcerative colitis

**Figure 4:**
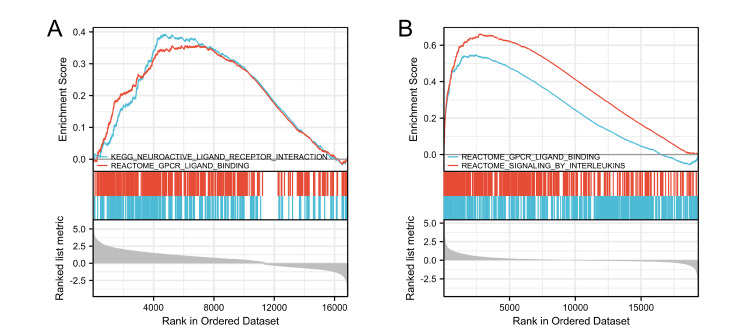
GSEA analysis of the datasets (A) GSEA of GSE35958 (OP) dataset. (B) GSEA of GSE87466 (UC) dataset. GSEA: Gene Set Enrichment Analysis, OP: osteoporosis, UC: ulcerative colitis

In this study, a total of 2337 differentially expressed genes were identified in OP based on the criteria of adjusted P-value < 0.05 and an |logFC| ≥ 1.0 (Figure 2A). Similarly, 1661 differentially expressed genes were identified in UC (Figure 2B). The Metascape database was employed to conduct GO and KEGG enrichment analyses for both OP and UC (Table 2). For OP, the results of GO and KEGG enrichment analyses are depicted in Figures 3A-3D. The primary BP involved positive regulation of cellular protein localization, positive regulation of intracellular protein transport, and regulation of intracellular protein transport. CC mainly targeted focal adhesion, cell-substrate adherens junction, and cell-substrate junction. MF primarily involved transcription coactivator activity, cadherin binding, and cell adhesion molecule binding. KEGG analysis for OP revealed that the main pathways involved were Adherens junction, Focal adhesion, and Proteoglycans in cancer. For UC, the GO and KEGG enrichment analyses are shown in Figure 3E-3H. The main aspects of BP included leukocyte migration, organization of the extracellular matrix, and organization of the extracellular structure. CC primarily targeted the collagen-containing extracellular matrix, the plasma membrane's exterior, and the secretory granule membrane. MF mainly involved glycosaminoglycan binding, structural components of the extracellular matrix, and cytokine activity. KEGG analysis for UC indicated that the primary pathways were rheumatoid arthritis (RA), viral protein interaction with cytokine and cytokine receptors, and cytokine-cytokine receptor interaction. Additionally, Figure 4 presents the results of the GSEA enrichment analysis for both OP and UC. This comprehensive analysis provides insights into the biological functions and pathways associated with the differentially expressed genes in OP and UC, contributing to a better understanding of the underlying mechanisms of these diseases.

**Table 2:** GO and KEGG analyses of GSE35958 and GSE87466. KEGG: Kyoto Encyclopedia of Genes and Genomes, GO: Gene Ontology, DEGs: differential genes

DEGs	Ontology	ID	Description	Count	p.adjust
GSE35958	BP	GO:0033157	regulation of intracellular protein transport	61	7.35513E-12
	BP	GO:0090316	positive regulation of intracellular protein transport	47	1.71622E-11
	BP	GO:1903829	positive regulation of cellular protein localization	76	5.05755E-11
	CC	GO:0005925	focal adhesion	117	9.1193E-22
	CC	GO:0005924	cell-substrate adherens junction	117	9.13433E-22
	CC	GO:0030055	cell-substrate junction	117	1.51679E-21
	MF	GO:0050839	cell adhesion molecule binding	112	1.57448E-09
	MF	GO:0045296	cadherin binding	81	1.16719E-08
	MF	GO:0003713	transcription coactivator activity	74	7.48203E-07
	KEGG	hsa04510	Focal adhesion	59	5.60412E-09
	KEGG	hsa04520	Adherens junction	29	9.47447E-08
	KEGG	hsa05205	Proteoglycans in cancer	56	1.2674E-07
GSE87466	BP	GO:0050900	leukocyte migration	115	9.03478E-27
	BP	GO:0030198	extracellular matrix organization	93	1.43215E-24
	BP	GO:0043062	extracellular structure organization	100	2.23921E-24
	CC	GO:0062023	collagen-containing extracellular matrix	94	5.79425E-23
	CC	GO:0009897	external side of plasma membrane	76	8.33238E-14
	CC	GO:0030667	secretory granule membrane	62	9.53361E-13
	MF	GO:0005539	glycosaminoglycan binding	57	1.02347E-13
	MF	GO:0005201	extracellular matrix structural constituent	44	7.09352E-12
	MF	GO:0005125	cytokine activity	51	2.98427E-11
	KEGG	hsa05323	Rheumatoid arthritis	35	5.20137E-12
	KEGG	hsa04061	Viral protein interaction with cytokine and cytokine receptor	35	3.29002E-11
	KEGG	hsa04060	Cytokine-cytokine receptor interaction	63	1.291E-09

Identify co-expressed differential genes

**Figure 5:**
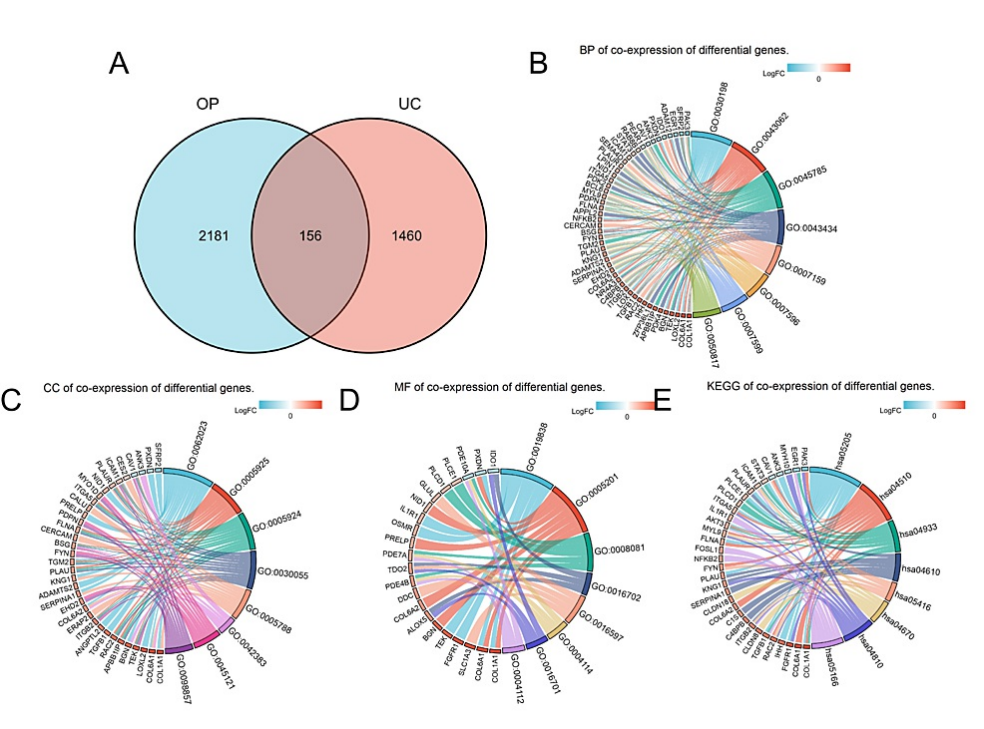
Screening and analyzing for co-expression differential genes (A) Venn of GSE35958 (OP) and GSE87466 (UC). (B) BP of co-expression of differential genes. (C) CC of co-expression of differential genes. (D) MF of co-expression of differential genes. (E) KEGG of co-expression of differential genes. OP: osteoporosis, UC: ulcerative colitis, BP: biological processes, CC: cellular components, MF: molecular function, KEGG: Kyoto Encyclopedia of Genes and Genomes

Utilizing the Venn 2.0 database, this study identified a total of 156 co-expressed differential genes shared between OP and UC (Figure 5A). Subsequent GO and KEGG enrichment analyses were conducted on these co-expressed differential genes (Figures 5B-5E) (Table 3). In terms of BP, the co-expressed differential genes were primarily involved in the organization of the extracellular matrix, organization of the extracellular structure, and positive regulation of cell adhesion. For CC, the genes were mainly associated with focal adhesion, cell-substrate adherens junction, and collagen-containing extracellular matrix. In the MF category, the genes were predominantly involved in growth factor binding, serving as extracellular matrix structural components, and exhibiting phosphoric diester hydrolase activity. KEGG analysis revealed that the co-expressed differential genes were most significantly involved in signaling pathways such as Proteoglycans in cancer, Focal adhesion, and the AGE-RAGE signaling pathway in diabetic complications. This analysis of co-expressed differential genes provides valuable insights into the shared biological functions and pathways between OP and RA, which can be instrumental in understanding the common mechanisms underlying these diseases and potentially developing targeted therapeutic strategies.

**Table 3:** GO and KEGG analyses of co-expression of differential genes. KEGG: Kyoto Encyclopedia of Genes and Genomes, GO: Gene Ontology

Ontology	ID	Description	p.adjust
BP	GO:0030198	extracellular matrix organization	5.94e-07
BP	GO:0043062	extracellular structure organization	2.93e-06
BP	GO:0045785	positive regulation of cell adhesion	6.10e-06
BP	GO:0043434	response to peptide hormone	4.73e-04
BP	GO:0007159	leukocyte cell-cell adhesion	4.73e-04
CC	GO:0062023	collagen-containing extracellular matrix	4.34e-05
CC	GO:0005925	focal adhesion	0.008
CC	GO:0005924	cell-substrate adherens junction	0.008
CC	GO:0030055	cell-substrate junction	0.008
CC	GO:0005788	endoplasmic reticulum lumen	0.010
MF	GO:0019838	growth factor binding	0.063
MF	GO:0005201	extracellular matrix structural constituent	0.091
MF	GO:0008081	phosphoric diester hydrolase activity	0.091
MF	GO:0016702	oxidoreductase activity, acting on single donors with incorporation of molecular oxygen, incorporation of two atoms of oxygen	0.091
MF	GO:0016597	amino acid binding	0.091
KEGG	hsa05205	Proteoglycans in cancer	2.39e-05
KEGG	hsa04510	Focal adhesion	4.68e-04
KEGG	hsa04933	AGE-RAGE signaling pathway in diabetic complications	4.68e-04
KEGG	hsa04610	Complement and coagulation cascades	0.001
KEGG	hsa05416	Viral myocarditis	0.014

PPI network construction of differentially expressed genes

**Figure 6:**
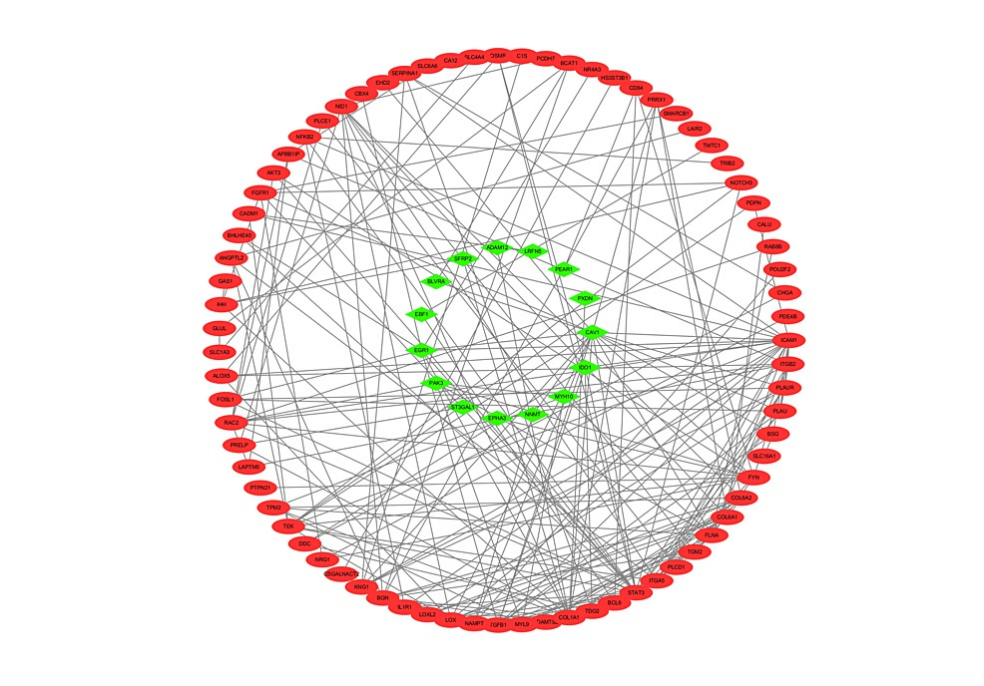
PPI network of co-expression of differential genes. Red is the upregulated gene. Green is the downregulated gene. PPI: protein-protein interaction

**Figure 7:**
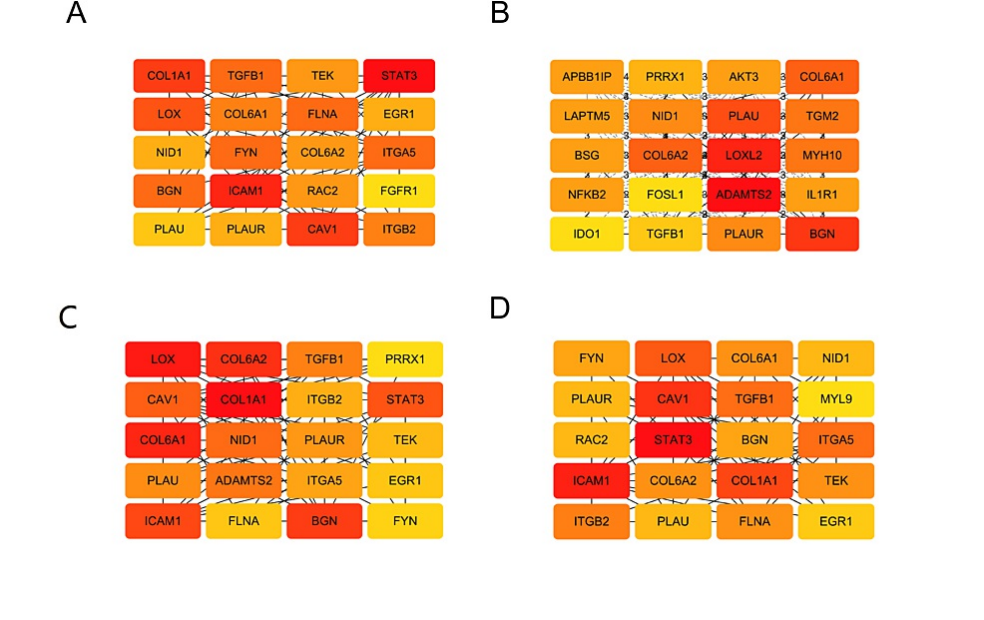
Hub genes based on four algorithms of Cytoscape 3.7.2 based on cytoHubba plugin. (A) Degree algorithm, (B) DMNC algorithm, (C) MCC algorithm, (D) MNC algorithm.

**Figure 8:**
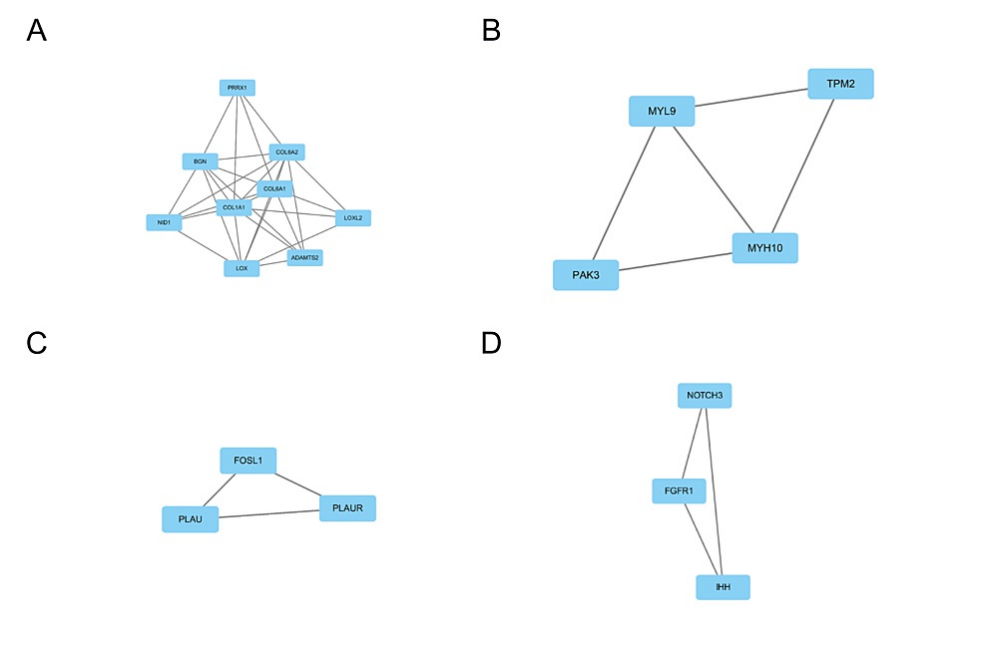
Screening for core module (A-D) The core module of Cytoscape 3.7.2 based on MCODE plugin.

**Figure 9:**
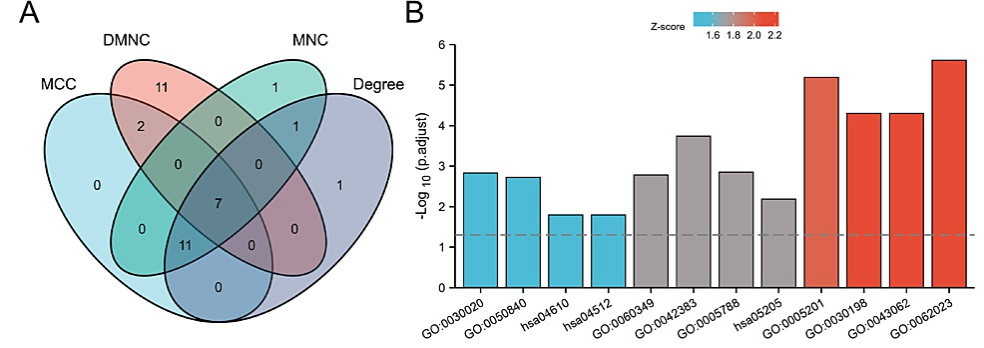
Screening and analyzing for hub genes. (A) Hub genes of Venn based on four algorithms of Cytoscape 3.7.2 based on cytoHubba plugin. (B) GO and KEGG of 7 Hub genes. KEGG: Kyoto Encyclopedia of Genes and Genomes, GO: Gene Ontology

The study employed the STRING 11.5 database and Cytoscape 3.7.2 software to construct and visualize the PPI network, which is depicted in Figure 6. The PPI network comprises 96 nodes and 222 edges, representing proteins and their interactions, respectively. To identify core modules within the PPI network, the MCODE plugin in Cytoscape 3.7.2 was used. The top four rated core modules were extracted and are displayed in Figure 7. Furthermore, the cytoHubba plugin in Cytoscape 3.7.2 was utilized to identify the top 20 hub genes using four algorithms: Degree, DMNC, MCC, and MNC. These are illustrated in Figure 8. Venn 2.0 was then used to select the overlapping genes common to the four algorithms, resulting in a total of seven hub genes, namely COL6A1, COL6A2, BGN, NID1, PLAU, TGFB1, and PLAUR (Figure 9A). The GO and KEGG enrichment analyses of these hub genes are presented in Figure 9B and Table 4. In terms of BP, the primary activities were bone morphogenesis, extracellular structure organization, and extracellular matrix organization. CC mainly involved the sarcolemma, endoplasmic reticulum lumen, and collagen-containing extracellular matrix. MF primarily consisted of extracellular matrix binding and structural constituents of the extracellular matrix that confer tensile strength. KEGG analysis revealed that the hub genes were significantly involved in pathways such as Proteoglycans in cancer, complement and coagulation cascades, and extracellular matrix (ECM)-receptor interaction. These findings are crucial as they highlight the key genes and pathways that may play a significant role in the pathogenesis of OP and UC, and can be potential targets for therapeutic interventions.

**Table 4:** GO and KEGG analyses of hub genes. KEGG: Kyoto Encyclopedia of Genes and Genomes, GO: Gene Ontology

Ontology	ID	Description	p.adjust
BP	GO:0030198	extracellular matrix organization	5.01e-05
BP	GO:0043062	extracellular structure organization	5.01e-05
BP	GO:0060349	bone morphogenesis	0.002
BP	GO:0070208	protein heterotrimerization	0.002
BP	GO:0002062	chondrocyte differentiation	0.002
CC	GO:0062023	collagen-containing extracellular matrix	2.42e-06
CC	GO:0042383	sarcolemma	1.82e-04
CC	GO:0005788	endoplasmic reticulum lumen	0.001
CC	GO:0005581	collagen trimer	0.003
CC	GO:0035579	specific granule membrane	0.003
MF	GO:0005201	extracellular matrix structural constituent	6.42e-06
MF	GO:0030020	extracellular matrix structural constituent conferring tensile strength	0.001
MF	GO:0050840	extracellular matrix binding	0.002
MF	GO:0034713	type I transforming growth factor beta receptor binding	0.023
MF	GO:0048407	platelet-derived growth factor binding	0.023
KEGG	hsa05205	Proteoglycans in cancer	0.007
KEGG	hsa04610	Complement and coagulation cascades	0.016
KEGG	hsa04512	ECM-receptor interaction	0.016
KEGG	hsa04974	Protein digestion and absorption	0.016
KEGG	hsa04510	Focal adhesion	0.049

Validation and construction of diagnostic models

**Figure 10:**
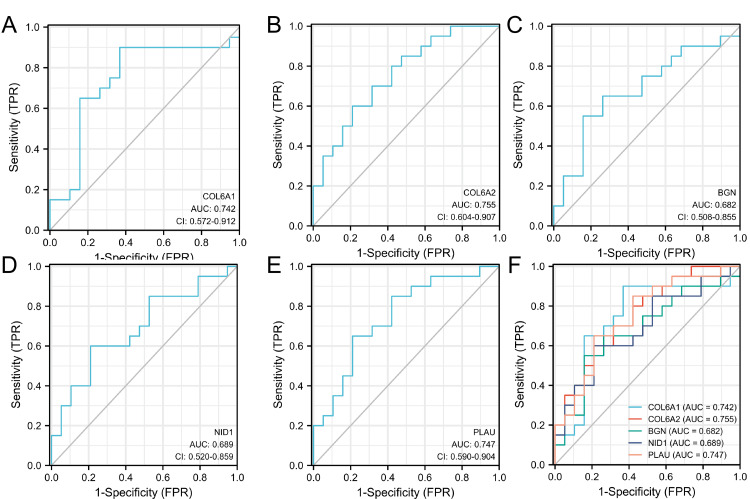
ROC validation of GSE56815 Diagnostic model of (A) COL6A1, (B) COL6A2, (C) BGN, (D) NID1, (E) PLAU, and (F) general map based on GSE56815 (OP). ROC: Receiver Operating Characteristic, OP: osteoporosis

**Figure 11:**
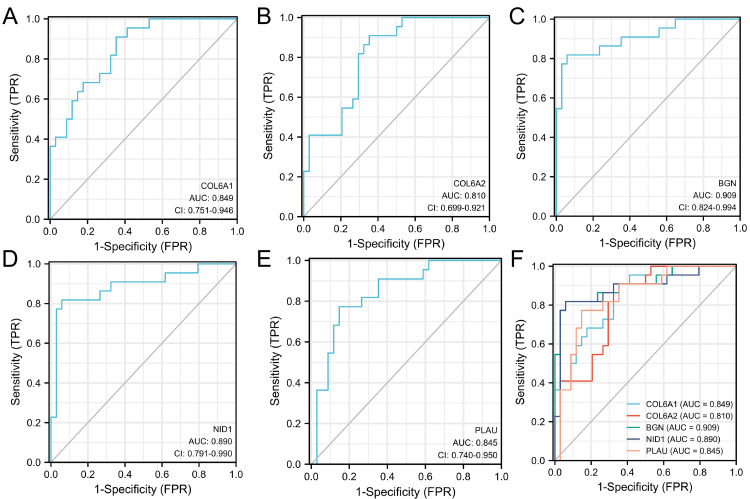
ROC validation of GSE107499 Diagnostic model of (A) COL6A1, (B) COL6A2, (C) BGN, (D) NID1, (E) PLAU, and (F) general map based on GSE107499 (UC). ROC: Receiver Operating Characteristic, UC: ulcerative colitis

The GSE56815 and GSE107499 datasets were used and the first five genes were selected for diagnostic model construction and validation. GSE56815(OP) results from a display (Figure 10), COL6A1 has an AUC of 0.742; COL6A2 has an AUC of 0.755; BGN has an AUC of 0.682; NID1 has an AUC of 0.689. PLAU has an AUC of 0.747; GSE107499 (UC) results from a display (Figure 11), COL6A1 has an AUC of 0.849; COL6A2 has an AUC of 0.810; BGN has an AUC of 0.909; NID1 has an AUC of 0.890; PLAU has an AUC of 0.845. These results suggest that these five genes are reliable targets for OP and UC.

## Discussion

OP and UC are conditions that often present with subtle early symptoms, making them difficult to diagnose in a timely manner. The global prevalence of both OP and UC is on the rise, posing a significant socioeconomic burden and causing distress and strain on the lives of patients. Interestingly, OP has been identified as one of the common extraintestinal complications of UC [11]. The prevalence of osteoporosis among patients with UC is increasing. While inflammation is believed to have a negative impact on bone metabolism, the exact mechanisms are not well understood. It is unclear whether bone loss in UC patients is a direct consequence of inflammation or if it is attributed to other factors such as poor mineral absorption, vitamin D deficiency, decreased physical activity, nutritional issues, or the use of medications like steroids and glucocorticoids. Research has indicated that inflammatory cytokines, such as TNF-α, can be produced in UC by activating T lymphocytes, which in turn can cause OP. Furthermore, TNF-α has been found to synergize with RANKL in acting directly on osteoclasts and their precursors. This regulates the OPG/RANKL/RANK pathway, leading to bone loss by promoting osteoclast production. In this study, 156 co-expressed differential genes were identified between OP and UC. Through the use of the cytoHubba plugin in Cytoscape 3.7.2, seven hub genes were pinpointed: COL6A1, COL6A2, BGN, NID1, PLAU, TGFB1, and PLAUR. Enrichment analysis revealed that these genes are primarily involved in the organization of the extracellular matrix, protein heterotrimerization, and chondrocyte morphogenesis in endochondral bone morphogenesis, among other biological processes. The identification of these hub genes and the understanding of their roles in biological processes can provide valuable insights into the mechanisms underlying the development of OP and UC. This, in turn, can pave the way for the development of more effective diagnostic tools and therapeutic interventions for these conditions.

Collagens are a family of proteins that play a crucial role in the structural integrity of various tissues in the body. COL6A1 (collagen type VI alpha 1) and COL6A2 (collagen type VI alpha 2) are members of the collagen family and are widely distributed in several tissues, including the skin, blood vessels, skeletal muscles, and lungs [12,13]. COL6A1 and COL6A2 contribute to the maintenance of the integrity of various tissues. Collagens, including COL6A1 and COL6A2, typically have a triple-helix domain, which is a common structural feature of these extracellular matrix proteins. Specifically, COL6A1 is a major structural component of microfibrils [14]. Mutations in the genes encoding COL6A1 and COL6A2 have been associated with certain diseases, including Bethlem Myopathy and Ullrich Congenital Muscular Dystrophy. These diseases are characterized by muscle weakness and joint abnormalities. Furthermore, COL6A1 and COL6A2 are involved in several biological pathways, including collagen chain trimerization and extracellular matrix organization. The extracellular matrix is essential for providing structural support to cells and tissues, and collagens like COL6A1 and COL6A2 are vital components of this matrix. Understanding the roles of COL6A1 and COL6A2 in tissue integrity and their involvement in diseases can be valuable for the development of therapeutic strategies for conditions associated with abnormalities in collagen and extracellular matrix [15,16].

BGN is an extracellular matrix protein that is encoded by the BGN gene. It is initially produced as a proprotein, which undergoes proteolytic processing to form the mature protein. BGN plays a crucial role in various biological processes, including bone growth, muscle development and regeneration, and the assembly of collagen fibers in different tissues [17,18]. In recent years, it has been hypothesized that BGN is a signaling molecule that mediates various stages of tumorigenesis [19]. Epithelial cell morphology, growth, differentiation, and migration are all mediated by BGN in a significant way. The primary function of BGN is to preserve the extracellular matrix's structural integrity [20]. BGN may have diagnostic and prognostic value for ovarian, prostate, gastric, and colorectal cancers, according to studies. This protein also regulates inflammation and innate immunity [21,22].

NID1 is a sulfated multifunctional glycoprotein widely found in basement membranes [23]. During inflammation, NID1 encourages the chemotaxis of neutrophils. Differentiation, proliferation, and apoptosis are all regulated by interactions between cells and basement membranes [24]. NID1 is expressed in various tissues and also binds to COL6A1 and Perlecan. It may play a role in cell-extracellular matrix interactions [25].

PLAU (Plasminogen Activator, Urokinase) is a Protein Coding gene. A secreted serine protease that converts plasminogen into plasmin is encoded by this gene [26]. The plasminogen activation system (PAS) is mainly involved in tissue remodeling and fibrinolysis and inflammation in various physiological states. PAS is most well-known for its ability to break down fibrin, but it is also upregulated in chronic inflammatory conditions like arthritis and atherosclerosis [27,28]. Studies have shown that various components of PAS are related to the pathophysiology of RA. Urokinase plasminogen activator (UPA), especially pro-inflammatory mediators, appears to play an important role in RA-associated bone and cartilage destruction [29]. Although we have integrated many bioinformatics methods and statistical methods, they still have their limitations. First, this is a retrospective study, so new clinical samples and data are lacking. Second, the identified genes were not verified by in vivo and in vitro experiments. In the end, the study is simply based on the GEO database. Data from other databases are not included.

## Conclusions

In this study, we performed multiple bioinformatics analyses and identified key target genes (COL6A1, COL6A2, BGN, NID1, and PLAU) for OP and UC. The treatment of OP and UC may have potential targets in these key target genes. As a result, additional insights into the early diagnosis and treatment of OP and UC may result from a more in-depth examination of these genes.
